# Psychometric Properties of the Dutch Version of the Eating Competence Satter Inventory (ecSI 2.0^TM^) in Community Adolescents

**DOI:** 10.3390/nu15214531

**Published:** 2023-10-25

**Authors:** Laurence Claes, Lore Vankerckhoven, Dirk Smits, Glenn Kiekens, Christina L. Robillard, Loes Stukken, Koen Luyckx

**Affiliations:** 1 Faculty of Psychology and Educational Sciences, Katholieke Universiteit Leuven, 3000 Leuven, Belgium; lore.vankerckhoven@kuleuven.be (L.V.); dirk.smits@odisee.be (D.S.); koen.luyckx@kuleuven.be (K.L.); 2 Child & Youth Institute, Katholieke Universiteit Leuven, 3000 Leuven, Belgium; 3 Faculty of Medicine and Health Sciences, University of Antwerp, Wilrijk, 2610 Antwerp, Belgium; 4 Research Department, Odisee University of Applied Sciences, 1000 Brussels, Belgium; 5 Center for Contextual Psychiatry, Katholieke Universiteit Leuven, 3000 Leuven, Belgium; 6 Department of Medical and Clinical Psychology, Tilburg University, 5037 AB Tilburg, The Netherlands; 7 Department of Psychology, University of Victoria, Victoria, BC V8P 5C2, Canada; christinarobillard@uvic.ca; 8 Eetexpert, Kessel-Lo, 3010 Leuven, Belgium; loes.stukken@eetexpert.be; 9 Educational Unit for Professional Training and Service in the Behavioural Sciences (UNIBS), University of the Free State, Bloemfontein 9301, South Africa

**Keywords:** eating competence, adolescents, reliability, validity, eating disorders, identity

## Abstract

Eating competence can help adolescents navigate their food choices and attitudes toward eating in a healthy and balanced way. In the present study, we investigated the psychometric properties of the Dutch translation of the Eating Competence Satter Inventory 2.0^TM^ (ecSI 2.0^TM^), which was developed to assess eating attitudes and behaviors. A sample of 900 Flemish adolescents completed the ecSI 2.0^TM DUTCH^ and two self-report measures on eating disorder symptoms and identity functioning (i.e., confusion and synthesis). Confirmatory factor analysis confirmed the four-factor structure of the ecSI 2.0^TM DUTCH^, and the resulting four subscales (i.e., Eating Attitudes, Food Acceptance, Internal Regulation, and Contextual Skills) showed acceptable-to-excellent reliability (αs ranging from 0.69 to 0.91). The ecSI 2.0^TM DUTCH^ also demonstrated scalar invariance across sex and age (<17 years, ≥17 years). Males reported significantly higher ecSI 2.0^TM DUTCH^ scores than females on the four subscales and the total scale. The two age groups did not significantly differ on the ecSI 2.0^TM DUTCH^ scales. Finally, scores on the ecSI 2.0^TM DUTCH^ subscales showed non-significant or small negative correlations with adolescents’ Body Mass Index (BMI), large negative correlations with eating disorder symptoms and identity confusion, and large positive associations with identity synthesis. The Dutch translation of the ecSI 2.0^TM^ is a valid and reliable instrument to assess eating competence skills in male and female adolescents.

## 1. Introduction

Adolescence is a critical period of development during which young individuals are susceptible to societal pressures, body image concerns, and changes in eating habits [1,2]. In addition to physical maturation processes, adolescents undergo psychological maturation processes and need to develop a personal identity. The development of eating competence can help adolescents to have a healthy relationship with food and their bodies. By learning to listen to their bodies, make informed food choices, and maintain a positive relationship with food, adolescents can establish healthy eating habits that extend into adulthood [3].

### 1.1. The Satter Eating Competence Model (ecSatter)

The Satter eating competence model (ecSatter) is an evidence- and practice-based conceptualization of the interrelated spectrum of eating attitudes and behaviors [3]. According to Satter [3] (p. S142), competent eaters are “positive, comfortable, and flexible with eating and are matter-of-fact and reliable about getting enough to eat of enjoyable and nourishing food”. The ecSatter model breaks down eating competence into four components: (1) attitudes about eating and food; (2) food acceptance skills; (3) internal regulation skills; and (4) skills and resources for managing the food context and orchestrating family meals [3] (p. S142). Positive eating attitudes refer to having a positive interest in food/eating, attunement to inner (e.g., appetite) and outer (e.g., food attractiveness and availability) food experiences, and self-trust about managing food [3]. Food acceptance skills refer to feeling comfortable in the presence of novel food and being willing to experiment with unfamiliar food (i.e., not being a “picky eater”) [3]. Internal regulation skills refer to the homeostatic processes of hunger, appetite, and satiety that maintain constitutionally appropriate body weight [3]. Dieting ignores these homeostatic mechanisms and triggers counterregulatory mechanisms that lead to weight gain [3]. Finally, contextual skills refer to engaging in structured meal planning and eating adequate amounts of preferred foods to satisfy hunger/appetite [3]. Maintaining a pattern of regular meals depends on three other competencies: a positive attitude toward eating, accepting food, and being attuned to signs of hunger, appetite, and satiety [3].

### 1.2. The Construction of the Eating Competence Satter Inventory (ecSI) and its Different Adaptations (ecSI, ecSI/LI, ecSI 2.0, ecSI 2.0^TM^)

The final version of the ecSI (ecSI 2.0^TM^) was developed in several steps, which are described below.

#### 1.2.1. ecSI

To assess the four components of eating competence, Satter developed the Eating Competence Satter Inventory (ecSI) [3]. The ecSI consists of 16 items divided over four subscales, capturing the four eating competencies: Eating Attitudes (five items), Food Acceptance (three items), Internal Regulation (three items), and Contextual Skills (five items). Each item is answered on a five-point scale with the response options always (score 3), often (score 2), sometimes (score 1), rarely (score 0), and never (score 0). The total ecSI score ranges from 0 to 48, and an ecSI total score ≥32 indicates eating competence [4]. The cut-off score of ≥32 was determined based on theoretical rationale (i.e., at least a minimum mean score of 4 (often) on the eating competence items) and the clinical experience of the original author. Lohse et al. [4] validated the ecSI in a community sample of 863 adults (78.7% female) and replicated the theoretical four-factor structure of the ecSI using exploratory factor analysis. Results showed that all four subscales were adequately reliable: Eating Attitudes (α = 0.84), Food Acceptance (α = 0.65), Internal Regulation (α = 0.75), and Contextual Skills (α = 0.74) [4]. Participants scoring higher on eating competence (ecSI total score ≥ 32) were generally older and reported lower Body Mass Index (BMI), fewer eating disorder symptoms, more physical activity, and more healthy food/meal choices [4]. Stotts and Lohse [5] investigated the test–retest reliability (interval two to six weeks) of the ecSI in 259 White females, which revealed moderate-to-high Spearman rank correlation coefficients: ecSI total score (*r_s_* = 0.68), Eating Attitudes (*r_s_* = 0.70), Food Acceptance (*r_s_* = 0.65), Internal Regulation (*r_s_* = 0.52), and Contextual Skills (*r_s_* = 0.70).

#### 1.2.2. ecSI/LI

In 2011, Krall and Lohse [6] investigated the validity of the ecSI in women with low income (*n* = 25). Four items of the ecSI were misinterpreted due to wording and clarity problems [6]. These four misinterpreted items were revised based on participants’ feedback, retested, and combined with the 12 unaltered items in the ecSI for Low-Income (ecSI/LI). Krall and Lohse [6] validated the ecSI/LI in 507 females with low income and showed that eating-competent women (ecSI/LI total score ≥ 32) reported more physical activity, more fruit and vegetable intake, better food planning, lower BMI, less body weight dissatisfaction, and fewer eating disorder symptoms compared to non-eating-competent women.

#### 1.2.3. ecSI 2.0

In 2015, Lohse [7] examined whether the ecSI/LI could also be used in the general population. She administered both the ecSI and the ecSI/LI (with the four revised items) to 127 participants; the correlation between both versions of the instrument was very high (*r* = 0.98). Accordingly, the author concluded that the ecSI/LI was also applicable to the general population and renamed the ecSI/LI as the Eating Competence Satter Inventory 2.0 (ecSI 2.0). In 2015, Tilles-Tirkkonen and colleagues [8] validated a preliminary Finnish translation of the ecSI 2.0 in a sample of 976 Finnish adolescents (54% female) aged 10–17 years old. They replicated the four-factor structure of the ecSI 2.0 using confirmatory factor analysis (CFA) and showed that the total scale and the four subscales were reliable: total ecSI 2.0 (α = 0.92), Eating Attitudes (α = 0.87), Food Acceptance (α = 0.78), Internal Regulation (α = 0.83), and Contextual Skills (α = 0.81). Eating-competent adolescents (ecSI 2.0 total score ≥ 32) reported higher levels of self-esteem and stronger identity coherence, were less dissatisfied with their body size, and had engaged in fewer attempts to reduce body weight. They also reported higher meal frequency, more consumption of fruits and vegetables, and more health-promoting family meals.

#### 1.2.4. ecSI 2.0^TM^

More recently, Godleski, Lohse, and Krall [9] investigated the factor structure of the ecSI 2.0 in 2010 community adults. The findings of the CFA confirmed the four-factor structure of the ecSI 2.0 but suggested that item 9 (i.e., “I trust myself to eat enough for me”) be moved from the Internal Regulation subscale to the Eating Attitudes subscale. This change improved the model fit and reduced the magnitude of the correlation between the Eating Attitudes and the Internal Regulation subscales (e.g., in sample A from *r* = 0.90 to *r* = 0.75). In sum, the final ecSI2.0^TM^ still consists of 16 items divided over four subscales, but with different item numbers: Eating Attitudes (n_items_ = 5 + 1 = 6), Food Acceptance (n_items_ = 3), Internal Regulation (n_items_ = 3 − 1 = 2), and Contextual Skills (n_items_ = 5). The correlational patterns of the ecSI2.0/ecSI2.0^TM^ (sub)scales with other variables remained very similar [9]. Given that the ecSI 2.0^TM^ is used both at the total and subscales level, Godleski et al. [9] also investigated a second-order factor model in which each of the subscales loaded on one higher-order latent factor (i.e., Eating Competence). The data fit this higher-order model well, and the subscales loaded on the overall higher-order factor as follows: Eating Attitudes (0.96), Food Acceptance (0.62), Internal Regulation (0.83), and Contextual Skills (0.82). Finally, de Queiroz and colleagues [10] investigated the validity and reliability of the Brazilian Portuguese (BR) version of the ecSI2.0^TM^ in adults. CFA confirmed the four-factor structure of the ecSI2.0^TM^ BR, and the internal consistency coefficients of the subscales were: ecSI2.0^TM^ BR total score (α = 0.87), Eating Attitudes (α = 0.79), Food Acceptance (α = 0.73), Internal Regulation (α = 0.53), and Contextual Skills (α = 0.82) [10].

### 1.3. Eating Competence in Adolescents

To our knowledge, no studies have investigated the psychometric features of the ecSI 2.0^TM^ in adolescents. Gaining insight into the eating competence skills of adolescents is important, however, given that these skills impact their (a) eating behaviors; (b) body attitude; and (c) identity development, which are closely related to each other [11,12]. Erikson described identity formation as a central developmental task of adolescence [13]. During their identity quest, adolescents may undergo an identity crisis characterized by a conflict between identity synthesis and confusion [13]. Successful identity development is characterized by the extent to which synthesis predominates confusion [13]. Adolescents scoring higher on identity synthesis experience self-continuity over time and place, and develop a stable set of goals. In contrast, adolescents higher on identity confusion experience a fragmented sense of self and struggle to find purpose in their lives [13,14]. A longitudinal study by Verschueren et al. [15] showed that identity confusion increased adolescents’ vulnerability to experiencing body dissatisfaction and disordered eating symptoms over time, whereas identity synthesis protected against these concerns. Additionally, their findings showed that body dissatisfaction and disordered eating symptoms positively predicted identity confusion and negatively predicted identity synthesis over time, showing that disordered eating symptoms can hamper normative identity development [15]. From previous studies, we also know that eating disorder symptoms are negatively associated with eating competence [4,6]. Thus, being able to assess and address deficits in eating competence in adolescents can help to promote a positive body attitude and synthesized identity. Indeed, a study by Tilles-Tirkkonen et al. [8] showed a positive association between eating competence and a coherent sense of self (i.e., identity synthesis).

Therefore, it is important to investigate whether the ecSI 2.0^TM^ is a valid and reliable instrument to assess eating competence in adolescents, including whether it is a measurement invariant across sex and age and predicts dysregulated eating behaviors, body attitude, and identity development [11,12]. 

### 1.4. The Present Study

To address the aforementioned gaps in the literature, we investigated four research questions within a sample of Flemish adolescents. First, we investigated the four-factor structure of the ecSI 2.0^TM DUTCH^ and the higher-order model with one latent construct (i.e., Eating Competence), as well as the correlations between the latent factors. Based on existing research, we expected to find support for a four-factor structure of the ecSI 2.0^TM DUTCH^, with a slightly better fit for a factor solution in which item 9 (i.e., “*I trust myself to eat enough for me*”) is placed within the Eating Attitudes subscale [9] compared to a second-factor solution in which item 9 is placed within the Internal Regulation subscale [7]. We also expected that the correlation between the Eating Attitudes and Internal Regulation subscales would be lower in the first-factor solution (*r* = ±0.75) compared to the second-factor solution (*r* = ±0.90), based on a study in adults [9]. We further hypothesized that a second-order model in which each of the subscales loaded on one higher-order latent factor (i.e., Eating Competence) would fit the data well [9]. Second, we examined the reliability of the total scale and the subscales. Based on prior research [10], we expected to find acceptable (α ≥ 0.70) internal consistency for the Food Acceptance subscale, good/excellent (α ≥ 0.80/90) internal consistency for the Contextual Skills and Eating Attitudes subscales, and poor internal consistency for the Internal Regulation subscale. Third, we examined the measurement invariance of the ecSI 2.0^TM DUTCH^ across sex and age. Given the lack of previous studies on the measurement invariance across sex and age of the ecSI 2.0^TM DUTCH^, no specific hypotheses were forwarded. Finally, we investigated the associations between the ecSI 2.0^TM DUTCH^ total/subscales and BMI, eating disorder symptoms (i.e., Drive for Thinness, Bulimia, and Body Dissatisfaction), and identity measures (i.e., diffusion, synthesis) to ascertain convergent/divergent validity. We hypothesized that the total ecSI 2.0^TM DUTCH^ and its subscales would be unrelated or negatively related to BMI and negatively associated with eating disorder symptoms [4,6] and identity confusion [8,11,12]. In contrast, we expected that the (sub)scales would be positively related to identity synthesis [8,11,12].

## 2. Materials and Methods

### 2.1. Participants and Procedure

In total, 2080 adolescents were invited to participate in the first wave of the IDentity and Embodiment in Adolescents—Longitudinal study (IDEAL-study). Of this sample, 923 adolescents (response rate = 44.38%) were willing to fill out the questionnaires and received parental informed consent (if they were younger than 16 years). Of the 923 adolescents, 23 adolescents did not fill out the ecSI 2.0^TM DUTCH^ and were excluded from the study. Of the remaining 900 adolescents, 58.8% (*n* = 529) identified as female, 40.8% identified as male (*n* = 367), 0.2% (*n* = 2) did not want to disclose their sex, and 0.2% (*n* = 2) did not answer the question. The latter four participants were excluded from the analyses examining the sex invariance of the ecSI 2.0^TM DUTCH^. The mean age of the 900 adolescents was 16.19 years (*SD_age_* = 1.31, range: 13–21 years; 97.55% ≤ 18 years; 2.44% > 18 years), and the mean BMI of the adolescents was 21.08 kg/m^2^ (*SD_BMI_* = 3.42, range: 14.34–42.44 kg/m^2^). To examine the age invariance of the ecSI 2.0^TM DUTCH^, we divided the sample into two age groups: an adolescent group (<17 years, range: 13–16 years, *n* = 500) and an emerging adulthood group (≥17 years, range: 17–21 years, *n* = 400).

The study took place in three secondary schools in Belgium between April and May 2022. The study was conducted in accordance with the Declaration of Helsinki and approved by the Social and Societal Ethics Committee of KU Leuven (protocol code G-2021-4436-R3[AMD] and date of approval 24 February 2022). Prior to data collection, all adolescents read and signed an informed consent form, with parental consent obtained for adolescents younger than 16 years. For older adolescents, parents were informed about the participation of their adolescents in the study. Adolescents filled out the questionnaires during school hours while researchers were present in the classroom to answer their questions. Completing the questionnaires took approximately one hour. After completing the questionnaires, the adolescents submitted them to the researchers in a sealed envelope. Adolescents who were absent on the day of data collection were invited via e-mail to fill out the questionnaire online on Qualtrics. To thank the adolescents for their participation in the study, they all received a movie ticket.

### 2.2. Instruments

#### 2.2.1. Eating Competence Satter Inventory 2.0^TM^-Dutch Version (ecSI 2.0^TM DUTCH^)

Eating competence was assessed using the ecSI 2.0^TM DUTCH^. The ecSI 2.0^TM DUTCH^ is the Dutch translation of the ecSI 2.0^TM^ [9], which was translated with the permission of Barbara Lohse and Ellyn Satter. The items were first translated into Dutch and then back-translated into English. The back-translated items were checked and approved by Barbara Lohse and Ellyn Satter. The ecSI 2.0^TM^ consists of 16 items rated on a five-point Likert scale. The 16 items are divided into four subscales: Eating Attitudes (n_items_ = 6), Food Acceptance (n_items_ = 3), Internal Regulation (n_items_ = 2), and Contextual Skills (n_items_ = 5) [9]. The psychometric evaluation of the ecSI2.0^TM DUTCH^ is the goal of the present study. Researchers wishing to use the ecSI 2.0^TM^ can contact the original author at https://www.ellynsatterinstitute.org/ecsi-2-0/ (accessed on 15 October 2023).

#### 2.2.2. BMI

To assess adolescents’ BMI, we requested their height in meters and their weight in kilograms. We calculated their BMI by dividing their weight in kilograms by their squared length in meters [weight in kilograms/(length in meters)^2^].

#### 2.2.3. Eating Disorder Symptoms 

To assess eating disorder symptomatology, we used the three eating disorder-specific subscales of the Eating Disorder Inventory-3 (EDI-3) and the Interoceptive Deficits scale, which have been validated in community samples [16,17]. Each of the 34 items of the ED-3 is rated on a six-point Likert scale ranging from 1 (never) to 6 (always). The Drive for Thinness subscale consists of seven items and measures a desire to be thinner and concerns about dieting, as well as weight preoccupation and fear of weight gain (e.g., ‘I’m thinking about going on a diet’; α = 0.93 in the present study). The Bulimia subscale consists of eight items and assesses binge eating and eating in response to negative emotions (e.g., ‘I eat when I’m upset’; α = 0.87 in the present study). The Body Dissatisfaction subscale consists of 10 items and measures dissatisfaction about the size and shape of particular body regions, such as buttocks, stomach, and hips (e.g., ‘I think my stomach is too fat’; α = 0.89 in the present study). Finally, the Interoceptive Deficits scale consists of nine items that focus on deficits in recognizing and reacting to emotional stimuli (e.g., ‘I have feelings that I can’t quite place’; α = 0.89 in the present study).

#### 2.2.4. Identity Functioning

To assess identity functioning, we administered the Erikson Psychosocial Stage Inventory (EPSI) [14]. The EPSI consists of 12 items rated on a five-point Likert scale, ranging from 1 (strongly disagree) to 5 (strongly agree). Six items measure identity confusion (e.g., ‘I feel mixed up’, α = 0.76 in the present study), and six items measure identity synthesis (e.g., ‘I know what kind of person I am’, α = 0.79 in the present study).

### 2.3. Analyses

First, we performed CFAs using Mplus version 8.8 [18] to test the four-factor structure of the ecSI2.0^TM DUTCH^ as described in Godleski et al. [9], the original model as described by Lohse [7], and a second-order model with the four subscales of eating competence loading on one higher-order latent construct (i.e., Eating Competence). Model parameters were estimated with the Weighted Least Square Mean and Variance (WLSMV) adjusted estimator because the data were ordinal. Two criteria were used to evaluate model fit: (1) the Comparative Fit Index (CFI) with values between 0.90 and 0.95 indicating acceptable fit and values > 0.95 indicating good fit; and (2) the Standardized Root Mean Square Residual (SRMR) for a relative fit with values < 0.08 indicating acceptable fit and values < 0.06 indicating good fit [19].

Second, we examined the reliability of the ecSI2.0^TM DUTCH^ total and subscales using Cronbach’s α coefficients. Alpha coefficients above 0.70, 0.80, and 0.90 were considered acceptable, good, and excellent, respectively [20].

Third, we investigated measurement invariance across sex and age (<17 years, ≥17 years). According to Chen [21], measurement invariance examines whether a questionnaire measures the same construct across different groups. Configural invariance examines whether each latent factor is associated with identical items across sex/age; metric invariance investigates whether factor loadings of items on the latent factor can be constrained to be equal across sex/age; and scalar invariance tests whether intercepts of latent factor indicators (i.e., items) can be constrained to be equal across sex/age [21]. To test for metric and scalar invariance, we relied on two fit indices: (1) the change in CFI (ΔCFI), for which values < 0.01 support measurement invariance and (2) the change in RMSEA (ΔRMSEA), for which values < 0.015 support measurement invariance [21]. If scalar invariance across sex/age is obtained, mean differences across sex/age groups can be meaningfully interpreted.

Fourth, we calculated Pearson correlation coefficients between the ecSI2.0^TM DUTCH^ total and subscales with BMI, eating disorder symptoms (i.e., Drive for Thinness, Bulimia, Body Dissatisfaction, and Interoceptive Deficits), and identity (i.e., diffusion and synthesis), with *r* < 0.20 referring to small effects, *r* between 0.20 and 0.30 referring to medium effects, and *r* > 0.30 referring to large effects [22].

## 3. Results

### 3.1. Factor Structure of the ecSI2.0^TM DUTCH^

Table 1 displays the fit indices of the different CFAs. The four-factor model with item 9 loading on the Eating Attitudes subscale [9] fit the data well (CFI = 0.967, SRMR = 0.045). Moreover, the original four-factor model with item 9 loading on the Internal Regulation subscale fit the data equally well (CFI = 0.968, SRMR = 0.044). Given that recent studies [9,10] used the factor structure with item 9 belonging to the Eating Attitudes subscale, we decided to use this model.

In Table 2, the standardized factor loadings of the items on each of the four subscales are displayed. All items had factor loadings above 0.60.

Table 3 shows the correlations between the four latent factors for both models. The correlation between the Eating Attitudes and Internal Regulation subscales was nearly identical to the original model of Godleski et al. (*r* = 0.88 vs. *r* = 0.91) [9]. Based on the high intercorrelation, we also fit a model in which the Eating Attitudes and Internal Regulation subscales collapsed (three-factor model; see Table 1). However, this model had a slightly worse fit to the data.

Additionally, we tested a second-order model in which the four subscales loaded on one higher-order latent factor (i.e., Eating Competence), which fit the data well (see Table 1). The factor loadings of each of the subscales on the higher-order latent factor were as follows: Eating Attitudes (0.92), Food Acceptance (0.63), Internal Regulation (0.94), and Contextual Skills (0.77), which resemble the findings of Godleski et al. [9]. The mean ecSI2.0^TM DUTCH^ total score was 30.39 (*SD* = 10.53), and 51.2% of the adolescents scored above the cut-off score of ≥32, meaning that they were considered competent eaters. 

Finally, model comparisons showed that the four-factor model (with item 9 belonging to the Eating Attitudes subscale) fit the data significantly better than the three-factor model (χ^2^_(3) difference test_ = 83.06, *p* < 0.001) and slightly better than the second-order model (χ^2^_(2) difference test_ = 39.41, *p* < 0.001). 

### 3.2. Reliability of the ecSI2.0^TM DUTCH^

Regarding the reliability of the ecSI2.0^TM DUTCH^, the Cronbach’s α coefficient for the total scale was 0.91, and the Cronbach’s α coefficients for the Eating Attitudes, Food Acceptance, Internal Regulation, and Contextual Skills subscales were 0.91, 0.69, 0.81, and 0.80, respectively.

### 3.3. Measurement Invariance of the ecSI2.0^TM DUTCH^ across Sex and Age

Table 4 displays the goodness-of-fit statistics for measurement invariance testing across sex of the ecSI2.0^TM DUTCH^. Changes in the CFI and RMSEA values between the configural, metric, and scalar models were below the cut-off values suggested by Chen [21], and as such, scalar invariance for sex was obtained.

Overall, we found significant sex differences for the four subscales [Wilks’ Lambda = 0.872, *F*(4, 891) = 32.688, *p* < 0.001, partial *ή*2 = 0.128] (see Table 5). Males scored significantly higher than females on the four ecSI2.0^TM DUTCH^ subscales, with a strong effect size for Eating Attitudes, a medium effect size for Internal Regulation, and small effect sizes for Contextual Skills and Food Acceptance. Moreover, males scored significantly higher than females on total Eating Competence. The mean total score of males (*M* = 34.44, *SD* = 8.50) was above the cut-off score for competent eating (total score ≥ 32), whereas this was not the case for females (*M* = 27.56, *SD* = 10.90).

Table 6 displays the goodness-of-fit statistics for measurement invariance testing across age groups (<17 years, ≥17 years) of the ecSI2.0^TM DUTCH^. Changes in the CFI and RMSEA values between the configural, metric, and scalar models were below the cut-off values suggested by Chen [21], and as such, scalar invariance for age was obtained. 

Overall, we did not find significant age differences for the four subscales [Wilks’ Lambda = 0.995, *F*(4, 895) = 1.133, *p* = 0.340, partial *ή*2 = 0.005] (see Table 7). Furthermore, we did not find a significant difference between both age groups for total Eating Competence. The mean total score of adolescents (<17 years; *M* = 29.95, *SD* = 10.72), as well as the mean total score for emerging adults (≥17 years; *M* = 30.92, *SD* = 10.27), were below the cut-off score for competent eating (total score ≥ 32).

### 3.4. Associations between the ecSI2.0^TM DUTCH^ and BMI, Eating Disorder Symptoms, and Identity

Finally, we correlated the ecSI2.0^TM DUTCH^ total and subscale scores with adolescents’ BMI, eating disorder symptoms, and identity (see Table 8). 

BMI showed small negative correlations with Eating Attitudes and the total score but no significant correlations with the other ecSI2.0^TM DUTCH^ subscales. Additionally, all subscales and the total score showed large, negative correlations with all eating disorder symptoms; higher scores on eating competence were associated with lower scores on Drive for Thinness, Bulimia, Body Dissatisfaction, and Interoceptive Deficits. All subscales and the total score also showed large positive correlations with identity synthesis, as well as large negative correlations with identity confusion. In sum, the ecSI2.0^TM DUTCH^ subscales were unrelated to BMI (except Eating Attitudes), negatively related to eating psychopathology, and positively associated with a sense of personal coherence. 

## 4. Discussion

The present study investigated the psychometric properties of the Dutch version of the ecSI2.0^TM^, which consists of four subscales: (1) Eating Attitudes; (2) Food Acceptance; (3) Internal Regulation; and (4) Contextual Skills. We replicated the four-factor structure of the ecSI2.0^TM^, as described by Godleski et al. [9], in a Flemish sample of adolescents. Unlike the four-factor structure of the ecSI2.0, we found that the ecSI2.0^TM DUTCH^ Internal Regulation subscale consisted of only two items (instead of three items). Having only two items to identify an underlying construct can be problematic, and we support the claim of Eisinga et al. [23] that using more items is ideal. Despite the low number of items for some subscales, however, we found that the four subscales had acceptable (Food Acceptance), good (Internal Regulation, Contextual Skills), and even excellent (Eating Attitudes) reliability, in line with the findings of Godleski et al. [9] and de Quieroz et al. [10]. The alpha coefficient was lowest for the Food Acceptance subscale, which is consistent with previous studies [4,5,8,9]. It is worth noting that some research has shown unacceptably low reliability for this subscale [5], and as such, it is important for future revisions of the ecSI2.0^TM^ to address this issue. The second-order model with one higher-order latent factor (i.e., Eating Competence), based on the subscales and items of the ecSI2.0^TM^, also fit the data well. This suggests that in addition to the subscale scores, we can also use the ecSI2.0^TM^ total score to measure overall eating competence in Flemish adolescents. Based on the ecSI2.0^TM^ total score (≥32), more than half of our sample of adolescents (51.2%) were considered eating-competent. This resembles the findings of Tilles-Tirkkonen et al. [8], who found that 58% of Finnish adolescents were considered eating-competent. 

Another key finding is that we obtained scalar invariance across sex and age (<17 years; ≥17 years) for the four factors of the ecSI2.0^TM DUTCH^. This suggests that mean differences in eating competence between groups can be meaningfully interpreted. On the one hand, we did not find significant age differences on the ecSI2.0^TM DUTCH^ subscales and total scores. On the other hand, males scored significantly higher on all ecSI2.0^TM^ subscales compared to females, which parallels the findings of other studies in college students [24,25,26]. However, one study showed that adolescent females were more eating-competent than adolescent males (aged 10–17 years) [8], and Lohse et al. [4] did not find a significant association between eating competence and sex in an adult population. Given these mixed findings, further studies are needed to clarify the role of sex in eating competence across developmental stages. One reason that females may report lower eating competence than males is that they tend to be more sensitive to appearance ideals and experience more body dissatisfaction than males [27,28]. Consequently, females may be more vulnerable to engaging in dieting behaviors, which prevent eating-competent behaviors (e.g., eating when feeling hungry, eating a variety of foods, and having regular eating patterns; [3]). Several studies have indeed shown that eating-competent attitudes and behaviors are negatively associated with disordered eating behaviors, which are also more prevalent among females [4,6]. In line with these findings, we also found strong negative associations between the ecSI2.0^TM^ subscales and disordered eating behaviors, such as drive for thinness, bulimia, body dissatisfaction, and interoceptive awareness deficits. Dysregulated eating behaviors such as dieting tend to lead to food restriction (i.e., lack of food acceptance); bulimic behaviors (e.g., binge eating) may disturb the internal regulation or homeostasis of hunger/satiety signals; and adolescents with dysregulated eating patterns often do not have positive eating attitudes or may lack contextual eating skills such as eating together with others [3]. 

Consistent with the literature, we found nonsignificant or small negative associations between adolescents’ BMI and the ecSI2.0^TM^ subscales [4,6,25,29]. This means that eating-competent adolescents tend to have a slightly lower body weight than their non-eating-competent peers. Furthermore, we showed that all ecSI2.0^TM^ subscales were strongly negatively related to identity confusion and positively related to identity synthesis. These findings are in line with Tilles-Tirkkonen et al. [8], who also showed that eating-competent adolescents report higher levels of self-esteem and sense of coherence. This is not surprising, given that identity synthesis is related to more positive body attitudes and fewer eating-disordered behaviors, whereas the opposite is found for identity confusion [11,12,30]. 

Taken together, our results demonstrate that the ecSI2.0^TM^ is a valid and reliable instrument to assess overall eating competence, as well as specific facets of eating competence (i.e., Eating Attitudes, Food Acceptance, Internal Regulation, and Contextual Skills) in Flemish adolescents. The instrument can be helpful in identifying adolescents who are struggling with their eating attitudes and behaviors. Additionally, the instrument can be used by medical doctors, dietitians, and mental health professionals to help adolescents struggling with weight issues reflect on their past and present weight, activity, food intake patterns, and eating competence [31]. Such reflection could help adolescents take a step toward improving their eating competence and potentially support their identity synthesis [31,32]. 

Several limitations should be acknowledged when interpreting the study findings. First, this is the first time that the ecSI2.0^TM^ was used in a Flemish sample of adolescents; future studies need to investigate the psychometric features of this instrument in other Flemish samples, including emerging adults and adults and participants who are struggling with weight- and body-related issues (e.g., eating disorders; obesity). Second, all variables were assessed with self-report measures, which can inflate the associations between the variables due to shared method variance. Therefore, future studies could also make use of a multi-method approach by objectively measuring anthropomorphic variables (e.g., weight and lengths), observing real-life eating behaviors, or using other informants (e.g., parents, teachers, and partners). Third, the data that we used were based on a cross-sectional design, so the directionality of effects between variables could not be determined (e.g., does identity confusion lead to lower eating competencies or vice versa?). Therefore, longitudinal studies are needed to determine the directionality of effects between variables and to investigate the co-development of these variables over time (e.g., eating competencies, body dissatisfaction, and eating-disordered behaviors). 

## 5. Conclusions

In a Flemish sample of adolescents, we found support for a four-factor structure of the Dutch version of the ecSI 2.0^TM^ (i.e., Eating Attitudes, Food Acceptance, Internal Regulation, and Contextual Skills). The four subscales showed acceptable-to-excellent reliability. The ecSI 2.0^TM DUTCH^ was also scalar invariant across sex and age (<17 years, ≥17 years). Males reported significantly higher scores than females on all ecSI 2.0^TM DUTCH^ subscales and the total Eating Competence score. The two age groups did not differ significantly on the ecSI 2.0^TM DUTCH^ subscales. Finally, the ecSI 2.0^TM DUTCH^ subscales and the total score showed no or small negative correlations with adolescents’ BMI, large negative correlations with eating disorder symptoms and identity confusion, and large positive associations with identity synthesis. In sum, results support the use of the ecSI2.0^TM DUTCH^ with Flemish youth, as it appears to be a valid and reliable measure of overall eating competence and four important facets of eating competence. We believe that the ecSI 2.0^TM DUTCH^ can potentially help to prevent and treat disordered eating behaviors in our youth.

## Figures and Tables

**Table 1:** Results of the confirmatory factor analyses on the ecSI 2.0^TM DUTCH^ items.

Model	CFI	SRMR	*χ* ^2^	df	RMSEA	90% CI RMSEA
First-order four-factor model with item 9 loading on EA [9])	0.967	0.045	836.605	98	0.092	0.086–0.097
First-order four-factor model with item 9 loading on IR	0.968	0.044	816.486	98	0.090	0.085–0096
First-order three-factor model with items of EA and IR loading on one factor	0.962	0.047	936.681	67	0.096	0.090–0102
Second-order model with the four ecSI2.0^TM^ subscales loading on one higher-order factor	0.965	0.049	897.362	100	0.093	0.087–0099

EA = Eating Attitudes subscale; IR = Internal Regulation subscale; CFI = comparative fit Index; SRMR = standard root mean square residual; RMSEA = root mean square error of approximation; 90% CI = 90% confidence interval.

**Table 2:** Eating Competencies Satter Inventory (ecSI 2.0^TM^) and factor loadings.

Items/First-Order Factors Loading on…	Factor Loadings
Eating Attitudes	
1. I am relaxed about eating	0.84
2. I am comfortable about eating enough.	0.89
4. I feel it is okay to eat food I like.	0.80
8. I am comfortable with my enjoyment of food and eating.	0.92
9. I trust myself to eat enough for me.	0.89
14. I enjoy food and eating.	0.80
Food Acceptance	
5. I experiment with new food and learn to like it.	0.65
6. If the situation demands, I can make do by eating foods I do not much care for.	0.70
7. I eat a wide variety of foods.	0.78
Internal Regulation	
10. I eat as much as I am hungry for.	0.90
13. I eat until I feel satisfied.	0.86
Contextual Skills	
3. I have regular meals.	0.68
11. I tune into food and pay attention to eating.	0.63
12. I make time to eat.	0.86
15. I consider what is good for me when I eat.	0.60
16. I plan for feeding myself.	0.93

**Table 3:** Correlations between the latent factor scores of the ecSI2.0^TM DUTCH^ subscales for the model with item 9 loading on EA (first line) and item 9 loading on IR (second line).

	Eating Attitudes	Food Acceptance	Internal Regulation
Eating Attitudes	-		
Food Acceptance	0.56 *** (0.55 ***)	-	
Internal Regulation	0.88 *** (0.91 ***)	0.49 *** (0.54 ***)	-
Contextual Skills	0.70 *** (0.67 ***)	0.59 *** (0.59 ***)	0.70 *** (0.74 ***)

*** *p* < 0.001, EA = Eating Attitudes, IR = Internal Regulation.

**Table 4:** Goodness-of-fit indices for testing measurement invariance across sex.

	*χ* ^2^	df	CFI	ΔCFI	RMSEA	ΔRMSEA
Configural	895.222	196	0.964		0.089	
Metric	874.584	208	0.965	0.001	0.085	−0.004
Scalar	926.942	236	0.964	−0.001	0.081	−0.004

df = degrees of freedom; CFI = comparative fit index; RMSEA = root mean square error of approximation.

**Table 5:** Means (standard deviations) of the ecSI2.0^TM DUTCH^ subscales for girls and boys.

	Females (*n* = 529)		Males (*n* = 367)			
	*M*	*(SD)*	*M*	*(SD)*	*F*(1, 894)	*Partial ή*2
Eating Attitudes	10.93	(5.27)	14.55	(3.63)	129.70 ***	0.127
Food Acceptance	5.22	(2.33)	5.84	(2.12)	16.65 ***	0.018
Internal Regulation	3.68	(1.89)	4.72	(1.46)	78.74 ***	0.081
Contextual Skills	7.72	(3.79)	9.33	(3.65)	40.27 ***	0.043
ecSI2.0^TM DUTCH^ Total Score	27.56	(10.90)	34.44	8.50	102.99 ***	0.103

*** *p* < 0.001. The reference values for effect sizes for Partial Eta Squared: small effect = 0.01; medium effect = 0.06; and large effect = 0.14.

**Table 6:** Goodness-of-fit indices for testing measurement invariance across age.

	*χ* ^2^	df	CFI	ΔCFI	RMSEA	ΔRMSEA
Configural	908.987	196	0.969		0.090	
Metric	878.166	208	0.971	0.002	0.085	−0.005
Scalar	901.198	236	0.971	0.000	0.079	−0.006

df = degrees of freedom; CFI = comparative fit index; RMSEA = root mean square error of approximation.

**Table 7:** Means (standard deviations) of the ecSI2.0^TM DUTCH^ subscales for age groups.

	Adolescents (<17 Years) (*n* = 500)		Emerging Adults (≥17 Years) (*n* = 400)			
	*M*	*(SD)*	*M*	*(SD)*	*F*(1, 898)	*Partial ή*2
Eating Attitudes	12.16	(5.10)	12.75	(4.84)	3.05	0.003
Food Acceptance	5.48	(2.17)	5.49	(2.39)	0.01	0.000
Internal Regulation	4.01	(1.83)	4.23	(1.74)	3.34	0.004
Contextual Skills	8.31	(3.78)	8.46	(3.84)	0.37	0.000
ecSI2.0^TM DUTCH^ Total Score	29.95	10.72	30.92	10.27	1.91	0.002

The reference values for effect sizes for Partial Eta Squared: small effect = 0.01; medium effect = 0.06; and large effect = 0.14.

**Table 8:** Correlations between the ecSI2.0^TM DUTCH^ total and subscale scores with BMI, eating disorder symptoms, and identity.

	Eating Attitudes	Food Acceptance	Internal Regulation	Contextual Skills	ecSI2.0^TM DUTCH^ Total Score
BMI	−0.18 **	−0.05	−0.06	−0.08	−0.14 **
EDI					
Drive for Thinness	−0.75 **	−0.23 **	−0.54 **	−0.36 **	−0.63 **
Bulimia	−0.52 **	−0.18 **	−0.35 **	−0.33 **	−0.46 **
Body Dissatisfaction	−0.66 **	−0.26 **	−0.47 **	−0.39 **	−0.59 **
Interoceptive Deficits	−0.59 **	−0.27 **	−0.46 **	−0.43 **	−0.57 **
EPSI					
Identity Confusion	−0.47 **	−0.19 **	−0.35 **	−0.36 **	−0.45 **
Identity Synthesis	0.46 **	0.21 **	0.35 **	0.38 **	0.46 **

BMI = Body Mass Index (*n* = 890); EDI = Eating Disorder Inventory-3 (*n* = 897); EPSI = Erikson Psychosocial Stage Inventory (*n* = 889). ** *p* < 0.001.

## Data Availability

Data are available upon request at laurence.claes@kuleuven.be.
